# Compact Chromatic Confocal Lens with Large Measurement Range

**DOI:** 10.3390/s24165122

**Published:** 2024-08-07

**Authors:** Ning He, Huiqin Hu, Zhiying Cui, Xinjun Xu, Dakai Zhou, Yunbo Chen, Puyin Gong, Youhua Chen, Cuifang Kuang

**Affiliations:** 1 Ningbo Research Institute, Zhejiang University, Ningbo 315100, China; 2 College of Optical Science and Engineering, Zhejiang University, Hangzhou 310058, China; 3 Ningbo Yongxin Optics Co., Ltd., Ningbo 315048, China

**Keywords:** spectral confocal sensor, dispersive objective lens, displacement sensor, spectrometer

## Abstract

Spectral confocal sensors are effective for measuring displacements. The core of the spectral confocal measurement system is a dispersive objective lens that uses optical dispersion to establish a one-to-one correspondence between the focusing position and wavelength, achieving high-resolution measurements in the longitudinal direction. Despite significant progress in dispersive objective lenses for spectral confocal sensor systems, challenges such as a limited dispersion range, high cost, and insufficient measurement accuracy persist. To expand the measurement range and improve the accuracy of the spectral confocal sensor, we designed a compact, long-axial dispersion objective lens. This lens has a simple structure that requires only six lens elements, two of which form cemented doublets. The system length is 58 mm, with a working distance of 46 ± 6 mm and a dispersion range of 12 mm within the wavelength range of 450–656 nm. The lens has an object-side numerical aperture (NA) of 0.22 and an image-side NA between 0.198 and 0.24, ensuring high light energy utilization. Finally, a spectral confocal measurement system was constructed based on the designed dispersive objective lens, and performance evaluation tests were conducted. The test results showed that the system achieved a resolution of 0.15 μm and a maximum linear error of ±0.7 μm, demonstrating high-precision measurement capabilities. The proposed lens design enables the development of more portable and cost-effective spectral confocal sensors.

## 1. Introduction

With the rapid development of micro- and nanoscale precision manufacturing in recent years, various precision measurement techniques have been widely adopted in the aerospace, automotive, and microelectronics industries. Optical measurements, which are known for their noncontact nature, fast response, and high accuracy, have been extensively utilized. Common optical measurement techniques include interferometry, triangulation, and spectral confocal measurements. Interferometry can provide high-precision information regarding the surface shape and film thickness [1,2,3]; however, it requires stringent environmental conditions and device stability, which limits its applicability to some materials. Triangulation can achieve long-distance measurements but requires a visible target surface and specific geometric conditions [4]. Additionally, optical sensors based on the FDTD (Finite-Difference Time-Domain) method [5] can simultaneously assess multiple parameters, such as the displacement, thickness or relief profile, tilt angle, and refractive index. Moreover, they achieve high precision and multifunctional measurements, but their measurement range is relatively small. In contrast, spectral confocal measurements are largely independent of the surface topography and offer a high resolution and sensitivity across diverse samples and environmental conditions. This technique accurately measures the surface displacement, thickness of transparent objects [6,7], and surface roughness or topography via one- or two-dimensional transverse scanning [8,9,10]. Therefore, spectral confocal measurements are particularly advantageous for applications requiring high accuracy and resolution, regardless of the surface conditions.

Spectral confocal sensors have been developed based on confocal microscopy [11] by incorporating wavelength-encoding technology. This approach uses optical dispersion to link the focusing position and wavelength, which enables the precise determination of the object’s axial position by analyzing the reflected light’s wavelength within the effective dispersion range. The dispersive objective lens is the core component of spectral confocal sensors, and its parameters, such as the dispersion range, dispersion distance, and numerical aperture, determine key performance indicators, such as the measurement range, working distance, and measurement inclination. In recent years, studies have optimized dispersive objective lenses to enhance the axial detection capabilities of these sensors. For example, Bai et al. [12] optimized a dispersive objective lens with a dispersion range of 400 µm within the wavelength range of 450–623 nm, and they achieved a calibration repeatability of ±0.3 µm. Jiang et al. [13] designed a dispersive objective lens with a measurement range of 1 mm and a calibration repeatability of ±0.3 µm, featuring an image space numerical aperture (NA) of 0.4436. Xie et al. [14] optimized a dispersive objective lens to achieve a dispersion range of 3.454 mm within the working wavelength of 450–700 nm. Li et al. [15] introduced a spectral confocal displacement sensor using a radial GRIN lens, and they achieved a dispersion range of 1215 µm within the operating wavelength of 420–620 nm. Liu et al. [16] addressed the limited measurement range issue by using a phase Fresnel strip as the dispersive objective lens. They achieved a dispersion range of 16 mm, an axial resolution of 0.8 µm, and a displacement measurement accuracy better than 0.4%; however, the proposed lens increased the system complexity and manufacturing cost. In addition to lens improvements, some studies focused on algorithms and system structures. For instance, Bai et al. [17] minimized the effects of light source variations and sample surface reflectivity changes by adding a reference arm with an x-shaped fiber optic coupler and a normalization algorithm. They achieved an axial displacement resolution of 1 µm over a dispersion range of 1.05 mm with a peak fitting error of less than ±0.2%. Liu et al. [18] proposed a spectral feature compensation algorithm and a peak wavelength extraction method based on Gaussian curve fitting. They used a segmented curve calibration algorithm to map the peak wavelength to the position, achieving a root mean square error (RMSE) of less than 0.1 µm for the displacement and less than 1 µm for the thickness within a dispersion range of 1.05 mm.

Despite significant progress in dispersive objective lenses for spectral confocal sensor systems, challenges such as a limited dispersion range, high costs, and insufficient measurement accuracy remain. Therefore, this study presents a compact dispersive objective lens with extended axial dispersion. We constructed a spectral confocal measurement system based on the designed dispersive objective lens and conducted performance evaluation tests. The results demonstrated that the system achieved a resolution of 0.15 µm and a maximum linear error of ±0.7 µm, thereby enabling high-precision measurements with good stability. In addition, the capability of the system for thickness measurement was verified.

## 2. Design of Dispersive Objective Lens

### 2.1. Spectral Confocal Measurement System Construction

**Figure 1:**
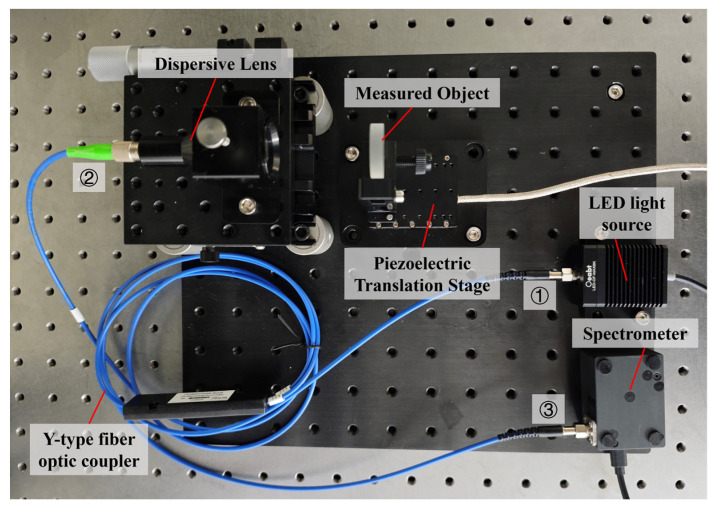
Spectral confocal measurement system.

The spectral confocal sensor operates based on the principle of optical dispersion to establish a direct relationship between the wavelength of light and its focused position. Figure 1 illustrates the spectral confocal measurement system, which comprises an LED light source, a Y-type optical fiber coupler with a 1:1 splitting ratio, a dispersive objective lens, and a spectrometer. In this study, the LED light source was positioned on the object side of the dispersive objective lens, while the object under test was located on the image side of the dispersive objective lens. The specific models of each component are listed in Table 1. Specifically, the core of the Y-type fiber coupler is 50 μm, and the NA of the fiber port is 0.22, which determines the object height and object-side numerical aperture during the design of the objective lens.

Light emitted from the LED source enters the system through branch ① of the Y-type fiber optic coupler and reaches the dispersive objective lens via branch ②. The dispersive objective lens separates the light into different wavelengths and focuses them at various positions along the optical axis. This establishes a one-to-one mapping relationship between wavelength and focal position, denoted as d=f(λ), where λ is the focused wavelength, f is the mapping function between wavelength and focal position, and d is the focal position corresponding to λ. The object being tested is placed within the dispersion range, and light reflected from its surface returns through branch ③, being captured by a spectrometer. Computer software is then used to analyze the spectral data, determining the wavelength λ of the reflected light using Gaussian fitting, thereby obtaining the precise positioning information d of the object’s surface.

We implemented several measures to mitigate stray light interference in the optical system. First, we applied a matte black coating inside the dispersive objective lens barrel. This coating absorbs excess light and prevents it from repeatedly reflecting within the barrel, thereby effectively reducing the impact of stray light on the measurement accuracy. Second, in terms of light transmission, the system employs fiber optic point-to-point transmission. Compared with structured light transmission [19], optical fibers avoid the noise issues caused by light scattering and reflection in free space. Finally, to further minimize reflective light interference within the fiber, we use FC/angled physical contact (APC) connectors with an 8-degree angled polish between the dispersive objective lens and the fiber. This design deflects the reflected light away from the fiber core, thereby increasing the return loss and significantly reducing the noise caused by the reflected light. To facilitate calibration and testing, the test object is securely mounted on a high-precision piezoelectric displacement stage to enable controlled movement within the dispersion range.

### 2.2. Design of Dispersive Objective Lens

The main performance indicators characterizing the spectral confocal sensor include measurement range, resolution, and maximum linear error. Based on the working principle of a spectral confocal system, the dispersive objective lens is the core of the spectral confocal sensor. The axial dispersion range of the dispersive objective lens determines the measurement range of the entire system. Increasing the dispersion range can expand the measurement range; however, it will reduce the light energy and lower the signal-to-noise ratio of the imaging. Increasing the numerical aperture on the image side can improve light utilization and imaging SNR; however, it also increases the difficulty of aberration correction. In addition, the lens design should include sufficient redundancy to facilitate assembly and adjustment. Therefore, when designing a dispersive objective lens, it is necessary to balance the various technical specifications to achieve optimal performance.

Assuming the dispersive objective lens consists of *N* lenses, the formula for calculating the dispersion range of the objective lens is [20]:

(1) $${\delta s^{\prime }}_{CH}=m^{2}{\delta s}_{CH}-f^{\prime 2}{\left(1-m\right)}^{2}\Sigma_{i=1}^{N}\frac{\varphi_{i}}{v_{di}}P_{Hi}$$

Here, ${\delta s^{\prime }}_{CH}=s_{H}^{\prime }-s_{C}^{\prime }$ and ${\delta s}_{CH}=s_{H}-s_{C}$; *s* is the distance of the object from the lens system, and $s^{\prime }$ is the distance of the image from the lens system, denoting H as the wavelength 450 nm. *C* represents the wavelength 656 nm; $f^{\prime }$ is the focal length of the optical system; *m* is the transversal magnification of the optical system; $\varphi_{i}$ are powers of individual lenses of the optical system; $v_{di}$ are Abbe numbers; and $P_{Hi}$ is relative dispersion.

We utilized the ZEMAX software to design and optimize the dispersive objective lens. We selected 450 nm, 585 nm, and 656 nm as reference wavelengths for the design. Based on the numerical aperture and core diameter of the Y-type fiber coupler, the object-side numerical aperture was set to 0.22, and the object height was set to 50 μm, resulting in a maximum field radius of 25 μm. The spectral confocal dispersive objective lens focuses solely on the size of the imaging spot and the beam’s focusing rather than image clarity, distortion, or astigmatism; hence, the primary aberration to correct is spherical aberration. Furthermore, according to the measurement principles of the spectral confocal system, retaining the chromatic aberration of the dispersive objective lens is necessary; therefore, during optimization, ZEMAX’s multi-configuration feature was used to evaluate the design using the size of the spot diagram on the best focal plane for the wavelengths 450 nm, 585 nm, and 656 nm. The smaller the spot size, the better the correction of spherical aberration, leading to a higher measurement accuracy. We employed optimization operands to comprehensively control the axial dispersion, effective focal length of the dispersive objective lens, center thickness of each lens, and edge thickness of each lens, aiming to achieve a dispersive objective lens that meets the performance requirements and is manufacturable.

**Figure 2:**
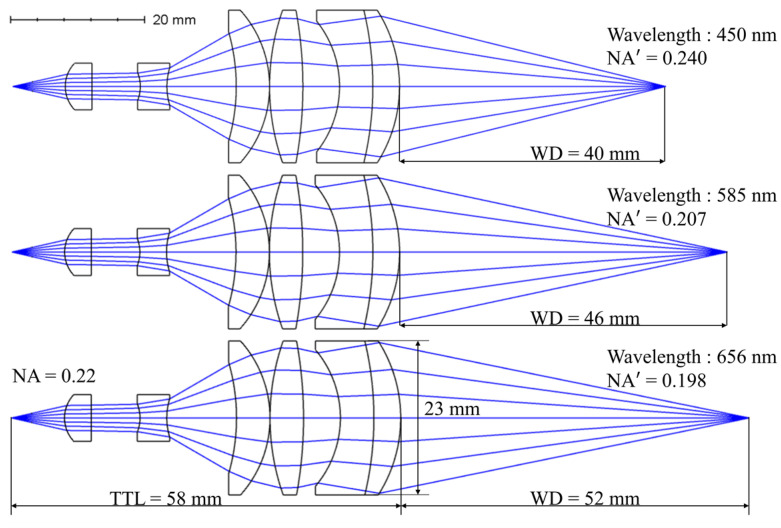
Light path at three design wavelengths.

The final optical path structure of the dispersive objective lens after design and optimization is shown in Figure 2; its technical specifications are listed in Table 2. The dispersive objective lens comprises six lenses, with a total track length (TTL) of 58 mm and a working distance (WD) of 46 ± 6 mm. The dispersion range within the working wavelength range of 450–656 nm is 12 mm. The image-side numerical aperture of the system is 0.198–0.240, which formed a 1:1 magnification with an object-side numerical aperture of 0.22, thus maximizing light utilization. The entire dispersive objective lens adopts a reverse telephoto structure, with a negative focal power group in the front and a positive focal power group in the rear. Table 3 presents parameters such as the radius, thickness, and clear semi-dia for each lens.

The first lens collimates the point light emitted by the optical fiber source from the object side to the image side of the dispersive objective lens. The second lens is a negative focal power group in the front, which diverges from the collimated light. The remaining four lenses of the dispersive objective lens formed a positive focal power group at the rear. The third and fourth lenses utilize high-refractive-index and low-Abbe-number glass materials to effectively increase the chromatic aberration of the system, thereby expanding the dispersion range. The fifth and sixth lenses form a doublet lens system that corrects the spherical aberration of the entire dispersive objective lens.

**Figure 3:**
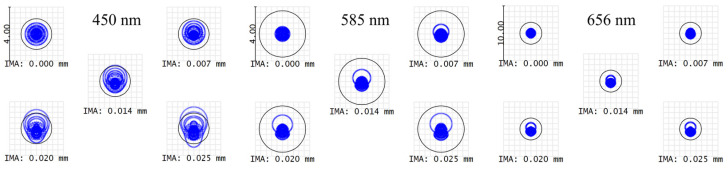
Spot diagram at three design wavelengths.

**Figure 4:**
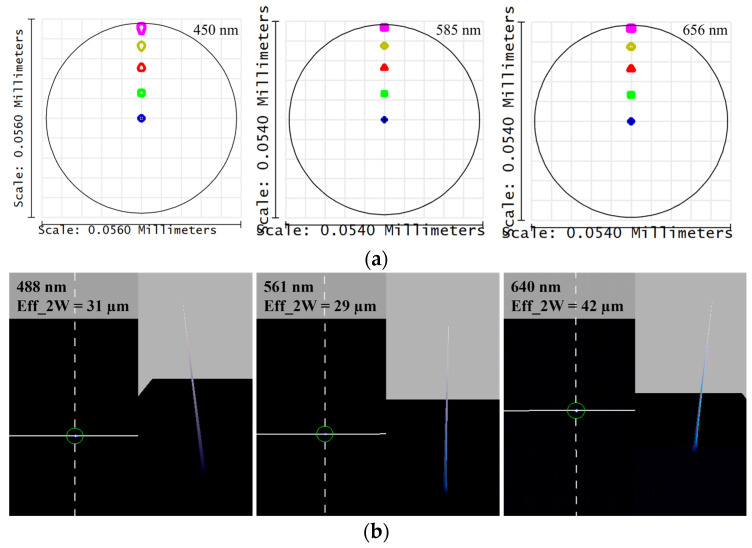
(**a**) Theoretical footprint diagram at different wavelengths; (**b**) practical footprint diagram at different wavelengths.

The size of the spot diagram can serve as a criterion for evaluating the focusing performance. A spot size smaller than that of the Airy disk indicates good spherical aberration correction and an excellent focusing performance. Figure 3 illustrates the spot diagrams at the best focal planes, corresponding to the wavelengths of 450 nm, 585 nm, and 656 nm, with their respective Airy disks. The spot sizes at these three wavelengths are smaller than or close to their corresponding Airy disks, achieving a diffraction-limited performance. Figure 4a depicts the theoretical ray trace diagram of the system, showing image heights of approximately 55 μm for different wavelengths, which is close to the 50 μm object height, consistent with a 1:1 magnification ratio of the system. Using fiber lasers at 488 nm, 561 nm, and 640 nm, light is coupled from a 9 µm fiber to a 50 µm fiber, and then the incident travels through the dispersive objective lens. The spot sizes at the focal point were measured using a beam quality analyzer (DataRay), as shown in Figure 4b, and all were less than 50 µm. Due to the difference in fiber core sizes, the field of view is not completely filled, resulting in a spot size smaller than 50 µm, which aligns with our measurement results.

**Figure 5:**
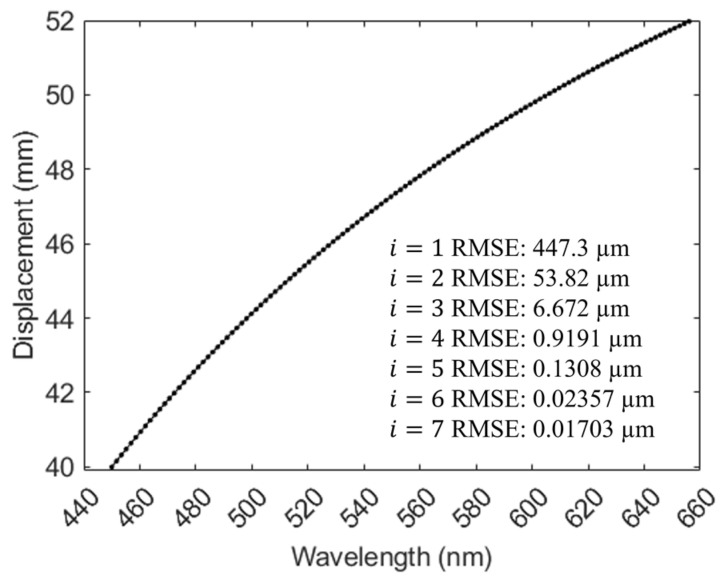
Theoretical relationship between the wavelength and the displacement, *i* stands for the polynomial fitting order, and RMSE stands for root mean squared error.

Figure 5 illustrates the theoretical focal displacement–wavelength curve of the system, which is nonlinear. The wavelength bandwidth was 450–656 nm, and the displacement range was 12 mm. Multiple fittings were performed in MATLAB. With fewer fittings, the root mean squared error (RMSE) was larger and did not meet the high-precision requirements. Conversely, excessive fitting reduces measurement speed. Therefore, interpolation was adopted for the calibration process in Section 3.

Light of different wavelengths focuses at different positions along the optical axis. The light intensity distribution at each position is given by [21]:

(2) $$I\left(\lambda_{i}\right)={\left[\frac{\pi a^{2}}{\lambda_{i}f^{\prime }(\lambda_{i})}sinc\left(\frac{\pi a^{2}b(\lambda_{i}-\lambda_{0})}{2\lambda_{i}f^{\prime 2}(\lambda_{i})}\right)\right]}^{4}$$

Here, $\lambda_{i}$ is any wavelength within the dispersive range, $\lambda_{0}$ is the central wavelength, a is the aperture of the dispersive objective, ${NA}^{\prime }=\frac{a}{2f^{\prime }}$, and b is the slope at the central wavelength in Figure 5. After neglecting the constant factor, we obtain the normalized light intensity distribution:

(3) $$I_{norm}\left(\lambda_{i}\right)={\left[sinc\left(\frac{2\pi b(\lambda_{i}-\lambda_{0}){NA}^{\prime 2}}{\lambda_{i}}\right)\right]}^{4}$$

Where $\lambda_{0}$ = 550 nm, b = 5680, and NA′ = 0.22, yielding a full width at half maximum (${FWHM}_{0}$) of 0.275 nm. The relationship between the theoretical axial resolution δz, dispersive range ${\delta s^{\prime }}_{CH}$, spectral range $P_{CH}$, and ${FWHM}_{0}$ of the system is:

(4) $$\frac{{\delta s^{\prime }}_{CH}}{\delta z}=\frac{P_{CH}}{{FWHM}_{0}}$$

According to Equation (4), the theoretical raw axial resolution of the system is 16 µm. Typically, sub-pixel peak detection algorithms can significantly enhance the peak detection accuracy [22]. Methods such as parabolic fitting, the centroid method, and Gaussian fitting are commonly used. In this system, we employ the Gaussian fitting method, which can improve the peak detection accuracy by approximately 100 times, thereby increasing the theoretical axial resolution of the system to 0.16 µm.

**Figure 6:**
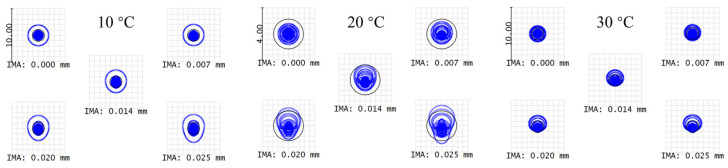
Spot diagram at different temperatures.

The dispersion objective of the spectral confocal displacement sensor is connected to the power and light sources via optical fibers to ensure that the system is not affected by temperature variations of the power and light sources. As a result, the system generally operates at around 20 °C and we designed it using a non-thermal approach. Nevertheless, we conducted a thermal analysis to evaluate the performance within the normal temperature range. Figure 6 shows that the spot diagram radius is comparable to the Airy disk size at temperatures ranging from 10 to 30 °C, confirming that the system’s performance remains within acceptable limits at around 20 °C.

To ensure that the dispersive objective lens maintains its excellent performance after manufacturing and assembly, we used ZEMAX software to evaluate the performance based on the root mean square (RMS) radius of the spot diagram. We employed a Monte Carlo simulation sensitivity analysis to assess the tolerance of the dispersive objective lens. Table 4 lists the tolerance distribution parameters we used, including the surface, element, and refractive index tolerances. Among these, the S + A irregularity accounts for all relevant tolerances in the optical system, including curvature radius tolerances, surface irregularities, and assembly errors. We conducted 100 Monte Carlo simulations, and the analysis results are presented in Table 5. The analysis indicates that, under the tolerance distribution conditions specified in Table 4, most of the simulated samples are expected to meet the design requirements after adjustments.

## 3. Results

**Figure 7:**
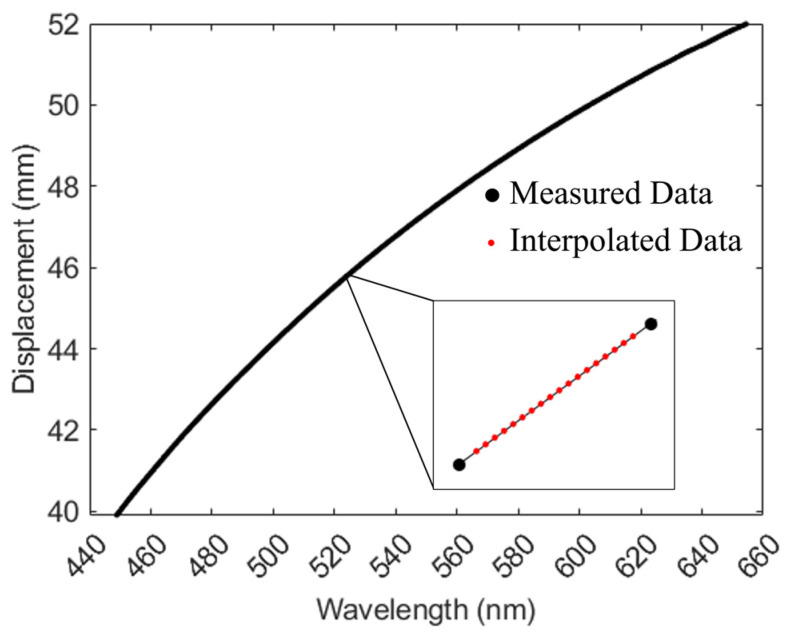
Practical relationship between wavelength and displacement.

Figure 1 shows the experimental setup. Before the measurement system begins operation, the relationship between the wavelength and focal position must be calibrated using a flat mirror as the test object. A flat mirror was mounted on a piezoelectric displacement stage with a linearity of 60 nm (Core Morrow N5630E-B1). The position of the test object was adjusted using a piezoelectric displacement stage with a displacement range of 12 mm within the 450–656 nm working wavelength band. The spectrometer captures the corresponding spectral data every time the piezoelectric displacement stage moves 20 μm. The center wavelength was obtained through Gaussian fitting, and the wavelength and position information were recorded after each move, resulting in 600 calibration datasets. As shown in Figure 7, these 600 datasets were then interpolated.

To verify the performance indicators of the spectral confocal sensor, we tested its resolution, maximum linear error, and thickness measurement capabilities.

### 3.1. Resolution

**Figure 8:**
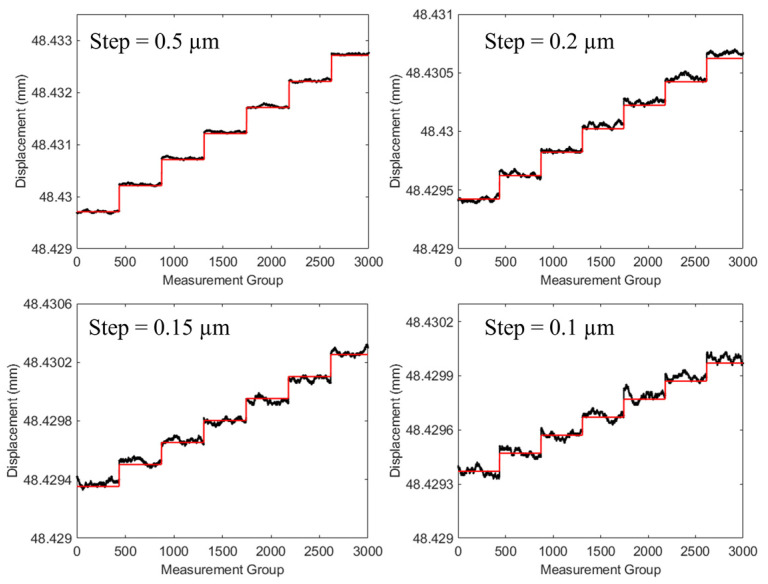
Resolution for step signals of different heights.

The axial resolution of the spectral confocal sensor indicated the ability of the system to discern the smallest displacement difference. In the resolution tests, we used step signals with heights of 0.5 μm, 0.2 μm, 0.15 μm, and 0.1 μm. The test object was a mirror, and the measurement results are shown in Figure 8. When the step height was 0.5 μm, the sensor could resolve it. For step heights of 0.2 μm and 0.15 μm, the measurement results showed fluctuations but no aliasing; therefore, they were still considered resolvable. However, when the step height was 0.1 μm, the signals from different steps exhibited aliasing and were considered unresolvable. Furthermore, the system’s resolution limit was precisely 0.15 μm at the wavelength with the poorest signal-to-noise ratio. Consequently, the resolution of the system across the entire measurement range was 0.15 μm.

### 3.2. Maximum Linear Error

**Figure 9:**
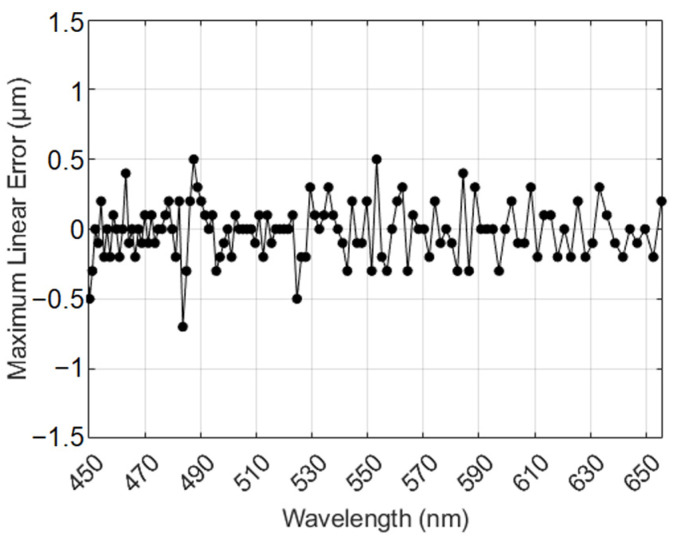
Maximum linear error at different wavelengths.

The maximum linearity error of the spectral confocal sensor reflects the measurement accuracy of the system. In the maximum linearity error test, we used a mirror as the test object and employed the piezoelectric platform to move the mirror within the dispersion range of the system. The displacement output by the piezoelectric platform was taken as the actual displacement value, and the difference between the system’s measured displacement value and the actual displacement value was considered to be the maximum linearity error. Figure 9 shows the relationship between the output wavelength and the maximum linearity error, indicating that the system’s maximum linearity error is less than ±0.7 μm.

### 3.3. Thickness Measurement

In thickness measurement, the upper and lower surfaces of the transparent object under test reflect light with wavelengths $\lambda_{1}$ and $\lambda_{2}$, respectively. According to the principle of spectral confocal microscopy and ignoring light refraction, the positions of the upper and lower surfaces of the measured object are given by $f(\lambda_{1})$ and $f\left(\lambda_{2}\right);$ the thickness is $f(\lambda_{2})$ − $f(\lambda_{1})$. However, light with wavelength $\lambda_{2}$ undergoes refraction before reaching the lower surface of the transparent object. Therefore, a refractive-index correction must be applied. The correction formula is as follows [7]:

(5) $$h_{2}=\frac{h_{1}\tan[\arctan({NA}^{\prime })]}{\tan\left\{\arcsin\left[\frac{n_{1}{\cdot NA}^{\prime }}{n_{2}}\right]\right\}}$$

Here, $h_{1}$ is the thickness of the measured object calculated without considering the refractive index factor, i.e., ${h_{1}=f\left(\lambda_{2}\right)-f(\lambda }_{1})$. ${NA}^{\prime }$ represents the image-side numerical aperture corresponding to different wavelengths of light. $n_{1}$ is the refractive index of light in air and $n_{2}$ is the refractive index of light in the measured object. As the image-side numerical aperture and the refractive index vary with different wavelengths, it is necessary to fit the numerical aperture ${NA}^{\prime }$ and the refractive index $n_{2}$ corresponding to the wavelength $\lambda_{2}$, and then substitute them into Equation (5) for calculation.

For thickness testing, we selected a Schott flat glass (BOROFLOAT 33) as the test object. The glass was fixed to a piezoelectric stage and positioned within the measurement range of the system. The measurement result from a coordinate measuring machine (Zeiss MICURA 575 with an error of 0.705 µm) is 1.9943 mm, which is taken as the reference value for the glass thickness.

**Figure 10:**
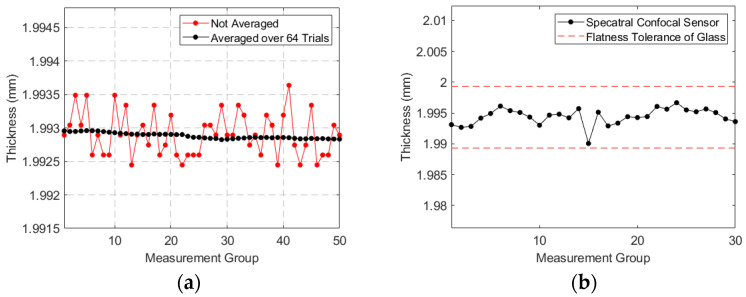
Results of thickness measurements: (**a**) 50 repeated measurements at the same position on the glass; (**b**) measurements at 30 different positions on the glass.

To verify the repeatability of the system thickness measurements, 50 measurements were conducted at the same location on the glass. By substituting the refractive index of the glass into Equation (5), we obtained the corrected thickness $h_{2}$, as shown in Figure 10a. The results indicate that the standard deviation of the initial repeated measurements was less than 1 µm. To reduce physical noise, most spectral confocal sensors average multiple measurements. We averaged 256 times, and the standard deviation of the thickness repeatability was found to be 0.15 µm.

To assess the ability of the system to measure the entire glass plate, we conducted thickness measurements at 30 different locations on the plate (Figure 10b). The results showed a variation of approximately ±3 µm across different locations. According to the datasheet, the flatness of SCHOTT float glass (BOROFLOAT 33) is less than 5 microns, suggesting that the thickness variation at different locations on the glass could be within 1.9943 ± 5 µm. This aligns closely with our measurement results, indicating that the system has high precision in thickness measurement.

**Figure 11:**
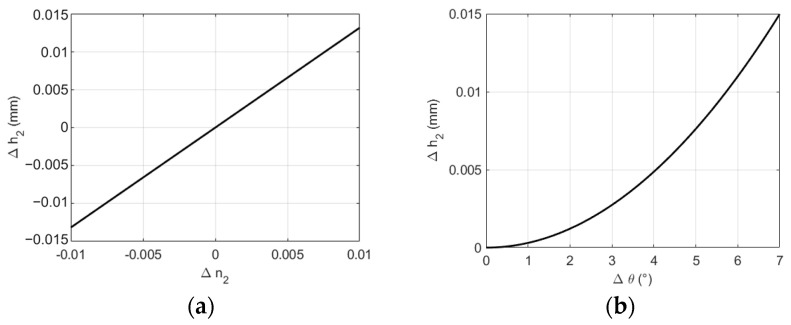
Influence of different factors on measurement results: (**a**) Influence of refractive index error on measurement results; (**b**) influence of the tilt angle of the measured object on measurement results.

When measuring the thickness of the glass, errors in the tilt between the dispersive objective lens and the measured glass, as well as errors in the refractive index of the measured glass, can affect the measurement results. These errors were quantified using Equation (1), as illustrated in Figure 11. $∆n_{2}$ is the error in the refractive index of the measured glass, ∆θ is the angle deviation between the cross-section of the dispersive objective lens and the measured object, and $∆h_{2}$ represents the variation in the thickness measurement results caused by these two factors. Both factors can influence the measurement results. Therefore, to improve the measurement accuracy when using this system for thickness measurements, it is advisable to input the refractive index tolerance and flatness of the measured object. Additionally, efforts should be made to ensure maximum horizontal alignment between the cross-section of the dispersive objective lens and the measured object to the extent possible.

## 4. Conclusions

Based on the principles of spectral confocal measurements, this study presented a compact dispersive objective lens designed with a long working distance and extended axial dispersion, achieving a dispersion distance of 12 mm within the 450–656 nm wavelength range. Each monochromatic wavelength reaches its diffraction limit at the focal point of the lens, ensuring a one-to-one correspondence between the object and image. The spot size at the final focal position was approximately 55 µm, demonstrating exceptional imaging quality and high measurement precision. Moreover, a spectral confocal measurement system was constructed using a Y-type optical fiber coupler. Calibration was performed using a precision piezoelectric stage with an accuracy of 60 nm. Interpolation was used to establish the relationship between the focal position and the wavelength. Several tests validated a resolution of 0.15 µm and a maximum linear error of less than ±0.7 µm. The system effectively measured the thickness of the transparent glass, as confirmed through repeated experiments that assessed its efficacy and analyzed potential factors influencing the results. The compact design of the dispersive lens favors its commercial application in spectral confocal imaging, whereas rigorous validation methods support the future design and testing of different models.

## Figures and Tables

**Table 1:** Equipment specification of the spectral confocal measurement system.

Equipment	Specification
Y-type fiber optic coupler	Core diameter: 50 μm Splitting ratio: 1:1 NA: 0.22
LED light source	LED-OF-MAX6K
Spectrometer	Flexible 4000 Resolution: 2.3 nm
Piezoelectric translation stage	CoreMorrow N5630E-B1

**Table 2:** Technical specifications of the dispersion objective lens.

Parameter	Value
Working wavelength	450–656 nm
Working distance	46 ± 6 mm
Axial dispersion	12 mm
Object-side NA	0.22
Image-side NA’	0.198–0.24

**Table 3:** Structural parameters of the optimized dispersion objective lens.

Surface	Radius	Thickness	Clear Semi-Dia
OBJECT	Infinity	3	0.027
1	Infinity	4.9	0.677
2	5.1	4	3.5
3	Infinity	7.26	3.5
4	−5.7	4	2.076
5	9.8	10.4	2.877
6	−30	5	8.278
7	−17.2	0	11.5
8	34.7	5	11.5
9	−63.85	5.5	11.5
10	−15.8	5	9.857
11	−49.4	4	11.5
12	−21.4	40	11.5
IMAGE	Infinity		0.027

**Table 4:** Tolerance allocation parameter table.

Tolerance Terms	Allocation Value
Thickness	0.01 mm
Surface tilt	0.3‘
Component tilt	0.3‘
Surface eccentricity	0.003 mm
S + A irregularity	0.1
Component eccentricity	0.01
Refractive index	0.0003
Aberration	0.03%

**Table 5:** Tolerance analysis results.

Wavelength	Radius of Airy Spot	90% Probability RMS in Monte Carlo Analysis
450 nm	1.143 μm	3.30110 μm
585 nm	1.714 μm	4.11116 μm
656 nm	2.003 μm	4.26328 μm

## Data Availability

The data that support the findings of this study are available from the corresponding author upon reasonable request.
